# Rumen-Protected Taurine Alleviates Heat Stress Injury in Hu Sheep by Regulating Inflammatory Response, Gut Microbiota and Transcriptome

**DOI:** 10.3390/ani16101445

**Published:** 2026-05-08

**Authors:** Yuting Wei, Yang Zhang, Xin Liu, Xinyu Chen, Guwei Lu, Yijie Wang, Xiaolong Hu, Kehui Ouyang

**Affiliations:** 1 Jiangxi Province Key Laboratory of Animal Nutrition, Animal Nutrition and Feed Safety Innovation Team, College of Animal Science and Technology, Jiangxi Agricultural University, Nanchang 330045, China; 2 Department of Food Science and Engineering, Jiangxi Vocational Technical College of Industry & Trade, Nanchang 330038, China

**Keywords:** heat stress, taurine, anti-inflammatory, gut microbiota, sheep

## Abstract

Prolonged high temperature and humidity pose significant challenges to sheep health and productivity due to heat stress. This research assessed the physiological regulatory impact of supplementing heat-stressed Hu sheep with rumen-protected taurine. Forty-eight animals were assigned to four groups receiving different taurine doses over a 63-day period. A comprehensive evaluation was conducted on growth performance, nutrient digestibility, serum biochemical parameters, antioxidant and immune indices, transcriptomic profiles, and gut microbiota composition. Dietary supplementation with 0.4% RPT significantly enhanced feed intake, daily weight gain, and feed efficiency. Serum analysis revealed increased taurine and high-density lipoprotein levels, reduced cortisol and inflammatory cytokines, and elevated antioxidant enzyme activity. Transcriptomic analysis identified differentially expressed genes enriched in immune and antiviral pathways, including JAK-STAT signaling pathway being among those highlighted. Gut microbiota profiling showed increased diversity and improved composition, particularly *Olsenella* and its several species were positively correlated with anti-inflammatory responses. These findings demonstrate that rumen-protected taurine supplementation at 0.4% can effectively alleviate heat stress by improving physiological resilience, immune balance, and gut health in sheep. This research provides a scientific basis for developing nutritional strategies to enhance animal welfare and productivity under climate stress conditions.

## Simple Summary

Summer high temperatures trigger heat stress in sheep, suppressing appetite, growth, and immunity. This study explored whether dietary supplementation with rumen-protected taurine (RPT) could help sheep cope better with heat stress. We fed different levels of RPT to sheep and monitored their growth, digestion and blood health, with gene activity and gut microbiota further determined only in the control and 0.4% RPT groups. The results showed that supplementation with 0.4% RPT effectively promoted growth performance and alleviated inflammatory responses in sheep. The 0.2% RPT treatment mainly improved fiber digestibility and serum antioxidant enzyme activities, while 0.6% RPT exerted the optimal anti-inflammatory effect but negatively affected serum immune status. At the molecular level, 0.4% RPT supplementation influenced key pathways related to stress and immunity. Meanwhile, it increased the presence of beneficial bacteria that may help protect against inflammation. This research suggests that RPT is a promising feed additive for alleviating heat stress and supporting the maintenance of health and productivity in sheep. Further research can focus on its mechanisms and explore its effects on other animals, helping farmers better manage heat stress in livestock.

## 1. Introduction

The rise in global climate change has significantly heightened the occurrence and persistence of extreme heat events, intensifying heat stress challenges in livestock production [1]. Heat stress not only disrupts thermal balance in animals but also triggers a series of physiological, metabolic, and immune disorders, becoming a major environmental stressor that limits the productivity and health of ruminants [2,3]. Research indicates that ruminants respond to ambient temperatures above their thermoneutral zone by enhancing respiratory rate, decreasing feed intake, and modifying blood flow distribution [4,5]. Nevertheless, such adaptive responses frequently lead to diminished growth performance, lower feed conversion efficiency, and heightened disease susceptibility [3,4,6]. Among ruminants, Hu sheep are widely raised in the midstream and lower parts of the Yangtze River and other warm regions of China due to their excellent reproductive performance and meat quality [7]. Nevertheless, Hu sheep are suffering heat stress under high-temperature environments in southern China frequently results in reduced average daily gain, lower feed efficiency, deterioration of carcass quality, and increased economic losses in production [8,9]. More importantly, heat stress disrupts rumen fermentation balance, impairs nutrient digestion and absorption, and damages intestinal barrier function, thereby inducing inflammatory responses and microbial dysbiosis [10,11]. These changes not only directly affect animal growth and health but may also exert long-term negative effects through immune and metabolic pathways. Consequently, devising strategies to alleviate heat stress impacts on Hu sheep’s growth and well-being in hot seasons is a pressing concern in animal nutrition and environmental physiology.

The small intestine is not only the primary site for nutrient digestion and absorption but also a critical barrier of the host immune barrier [12]. Tight junctions in intestinal epithelial cells preserve barrier integrity, stopping harmful pathogens and toxins from entering the body, while dynamically interacting with the gut microbiota to jointly regulate host metabolic and immune homeostasis [13]. Under heat stress conditions, however, the intestinal barrier function of ruminants is often compromised [14]. Elevated temperatures can compromise intestinal integrity by reducing mucosal blood flow, increasing epithelial cell apoptosis, and downregulating tight junction proteins. This enhances intestinal permeability, facilitating the translocation of endotoxins such as lipopolysaccharide (LPS) into the bloodstream, thereby triggering systemic inflammatory responses [14]. Heat stress significantly alters gut microbiota composition, decreasing beneficial bacteria such as fiber-degrading species and increasing potentially pathogenic bacteria [15]. Research indicates that gut microbiota and their metabolites can regulate inflammatory cytokine expression through various signaling pathways, affecting systemic immunity and metabolic equilibrium [16]. Consequently, impairment of intestinal health under heat stress is not merely a local issue but is closely linked to systemic metabolic disorders, immune suppression, and reduced production performance. Nutritional interventions targeting intestinal barrier function and microbial homeostasis may therefore represent an effective strategy to lessen the harmful effects of heat stress and sustain the health and productivity of ruminants.

Taurine is a sulfur-containing free amino acid derivative widely distributed in animal tissues, with particularly high concentrations in the heart, skeletal muscle, liver, brain, and immune organs [17]. Taurine, unlike most amino acids, is not directly integrated into proteins but serves various physiological functions such as maintaining cellular osmotic balance, regulating calcium levels, offering antioxidant protection, conjugating bile acids, stabilizing cell membranes, and modulating immune responses [18]. Research has indicated that taurine can scavenge oxygen and nitrogen species that are reactive, known as ROS and RNS, inhibit lipid peroxidation, attenuate inflammatory responses, and influence immune cell differentiation and function potentially through signaling pathways such as JAK-STAT, MAPK and NF-κB, although this requires direct experimental validation [19]. In ruminant nutrition research, the potential value of taurine has gradually attracted attention. However, due to degradation by rumen microorganisms, orally administered taurine is largely broken down before reaching the small intestine, resulting in markedly reduced bioavailability. The application of rumen-protected technology, which physically or chemically encapsulates taurine, can effectively prevent its degradation in the rumen, enhance its release and absorption in the small intestine, and thereby strengthen its physiological effects in the host. While prior research on yaks [20,21,22] and cattle [23,24] has demonstrated that rumen-protected taurine (RPT) enhances immune function, growth, and antioxidant capacity, its mechanistic effects under heat stress are not well understood.

While numerous studies have examined nutritional interventions for heat-stressed Hu sheep [25,26], the use of RPT in this area has not yet been investigated. Furthermore, the absence of an integrated analysis restricts a thorough understanding of RPT’s overall impact on heat-stressed Hu sheep. This study systematically evaluated the effects of dietary RPT supplementation on growth performance, physiological metabolism, antioxidant capacity, immune function, gut microbiota composition, and intestinal epithelial transcriptomic profiles in heat-stressed Hu sheep, while exploring potential molecular regulatory mechanisms. The results aim to offer theoretical and practical insights for managing the nutrition of Hu sheep experiencing heat stress.

## 2. Materials and Methods

### 2.1. Study Animals and Experimental Design

The experiment involved 48 healthy male Hu lambs, all 3 months old and with comparable body weights (26.93 ± 2.68 kg). The lambs were randomly assigned to four treatment groups, with 12 lambs per treatment group. During the experiment, all lambs were raised in individual pens. The pens were partitioned and retrofitted by adding isolation barriers, with each individual pen having a space of about 2 m × 1.5 m. The sheep were fed independently, and residual feed was collected individually. The supplemental levels of rumen-protected taurine (RP-Tau) were set at 0%, 0.2%, 0.4%, and 0.6% of the dry matter of the basal diet, corresponding to the following groups: The first group was offered the basal diet without RPT; the second group was provided with the basal diet supplemented with 0.2% RPT; the third group was provided with the basal diet supplemented with 0.4% RPT; the fourth group was provided with the basal diet supplemented with 0.6% RPT. The supplemental dosages of RPT in this experiment were determined by referring to previous studies on dairy cows and pigs, in combination with the heat stress challenge conditions [27,28,29].

The study was conducted from July to September 2023 at the Hu sheep breeding base in Ganzhou, Jiangxi Province. The trial consisted of a 7-day pre-experimental adaptation phase followed by a 63-day formal experimental period. RPT particles were thoroughly blended with concentrate and forage to formulate the total mixed ration (TMR), which was then fed to Hu sheep daily. Feeding was carried out twice daily at 08:00 and 17:00, and water was provided ad libitum. Management practices and immunization protocols were executed following the farm’s standard operating procedures.

Ambient temperature and relative humidity were continuously monitored during the experiment using a digital thermo-hygrometer (TH-179, Apresys Inc., Los Angeles, CA, USA). The temperature-humidity index (THI) was calculated using formula: THI = 0.8 × T + ((RH/100) × (T − 14.3)) + 46.4, where T represents the ambient temperature in degrees Celsius and RH denotes the relative humidity percentage [1]. Lambs were kept in shaded, well-ventilated pens without artificial cooling, allowing for natural heat stress exposure. During the experiment, Hu sheep were categorized by heat stress levels based on the Temperature-Humidity Index (THI): THI < 72 indicated no or mild pre-stress; THI ≥ 72 marked the onset of mild heat stress; THI ≥ 78 signified the onset of moderate heat stress; and THI ≥ 86 represented the onset of severe heat stress.

### 2.2. Experimental Diet Formulation

The study’s basal diet was formulated according to the Nutrient Requirements of Meat Sheep (https://www.chinesestandard.net/PDF/English.aspx/NYT816-2021, accessed on 4 May 2026), maintaining a concentrate-to-forage ratio of 60:40. Table 1 details the composition and nutrient profile of the basal diet. Taurine was purchased from Shandong Huayi Biotechnology Co., Ltd., Jinan, China, with a purity of 99.8%. The rumen-protected taurine (RP-Tau) was prepared by Kangdequan Biotechnology Co., Ltd. (Hangzhou, China), with the following characteristics: taurine content of 61.4%, rumen bypass rate of 86.3% at 12 h, and small intestinal release rate of 91.2% at 12 h. The coating material consists of cellulose acetate phthalate, styrene-maleic acid copolymer, and hydroxypropyl methylcellulose phthalate.

### 2.3. Sample Collection and Index Determination

#### 2.3.1. Determination of Growth Performance

Body weights of all experimental lambs (*n* = 12) were recorded before morning feeding on Day 1 and Day 64 (the final day) of the formal trial period. The weight measured on Day 1 was designated as the initial body weight (IBW), and the weight on Day 64 was recorded as the final body weight (FBW). Based on this data, the average daily gain (ADG) was calculated for the entire experimental periods. Additionally, before morning feeding each day, residual feed in the troughs was collected and weighed. Each animal’s daily feed consumption and any leftover feed were precisely documented. These data were utilized to determine the lambs’ average daily dry matter feed intake (ADFI) and feed-to-gain ratio (F/G). The calculation methods were: ADG (kg/d) = (FBW − IBW)/Experimental Days Count; ADFI (kg/d) = Total Dry Matter Intake of the Feed/Experimental Days Count; F/G = Total Dry Matter Intake of the Feed/Total Weight Gain.

#### 2.3.2. Collection and Analysis of Rumen Fermentation Parameters

On Day 57 of the formal trial, rumen fluid was sampled before the morning feeding with the help of an oral stomach tube sampler (*n* = 12). The initial 10 mL of effluent was discarded to eliminate salivary contamination. The subsequent rumen fluid was collected and immediately filtered through four layers of gauze. The pH of a portion of the filtrate was promptly measured using a portable pH meter (CT-6023, Kedida, Shenzhen, China). The filtrate was divided into 10 mL centrifuge tubes and stored at −20 °C for subsequent analysis of microbial protein (MCP), ammonia nitrogen (NH_3_-N), and volatile fatty acids (VFA). NH_3_-N concentrations were measured using the phenol–sodium hypochlorite colorimetric method [30]. MCP content was determined using a modified Lowry method according to Makkar et al. [31]. VFA concentrations were analyzed using a Shimadzu GC-2014 gas chromatograph, as per the methodology described by Wei et al. [32].

#### 2.3.3. Fecal Sample Collection and Determination of Apparent Nutrient Digestibility

During the experimental period, feed was sampled during the early, middle, and late stages of the trial (*n* = 12). After thorough homogenization, the samples were reduced using the quartering method. The subsamples were stored at −20 °C for later analysis. From Day 50 to Day 54 of the formal trial, a 5-day digestibility study was conducted. In each treatment group, six lambs were randomly selected for fecal sampling. Fecal samples (100 g each, *n* = 6) were collected from each lamb twice daily, both in the morning and evening. After collection, the samples from each lamb were thoroughly homogenized to obtain a representative composite sample. All feed and fecal samples were treated with 10% tartaric acid solution at a ratio of 1:4 (*m*/*v*) to fix nitrogen. The samples were dried at 65 °C until a constant weight was achieved. The dried samples were ground with a laboratory grinder and sieved through a 40-mesh to achieve a uniform powder. The powdered samples were kept in sealed bags for later analysis of nutrient digestibility and chemical composition.

Routine nutritional analysis of fecal and feed samples was conducted using standardized methods: dry matter (DM) content was measured following GB/T 6435-2014 [33]; neutral detergent fiber (NDF) and acid detergent fiber (ADF) were assessed according to GB/T 20806-2022 [34] and NY/T 1459-2022 [35], respectively; ether extract (EE) was determined via the Soxhlet extraction method as per GB/T 6433-2006 [36]; crude protein (CP) was analyzed using the Kjeldahl method in accordance with GB/T 6432-2018 [37]; total phosphorus was evaluated following GB/T 6437-2018 [38]; calcium content was measured according to GB/T 6436-2018 [39]; and acid-insoluble ash (AIA) was determined using GB/T 23742-2009 [40]. Nutrient apparent digestibility was determined using acid-insoluble ash (AIA) as an internal marker, applying the formula: Apparent Digestibility (%) = [1 − (F_2_/F_1_) × (A_1_/A_2_)] × 100. Here, A_1_ represents the AIA content in the feed (%), A_2_ the AIA content in the feces (%), F_1_ the target nutrient content in the feed (%), and F_2_ the target nutrient content in the feces (%).

#### 2.3.4. Serum Collection and Determination of Physiological and Biochemical Parameters

On the 60th day of the formal trial, a 20 mL blood sample was collected from the jugular vein using vacuum tubes, one hour prior to the morning feeding (*n* = 12). The blood was dispensed into 10 mL non-anticoagulant tubes and rotated at 3000 rpm for 20 min. The serum was aliquoted into 2 mL cryogenic tubes and stored at −20 °C for subsequent analysis. Serum levels of total cholesterol (CHO), triglycerides (TG), high-density lipoprotein (HDL), and low-density lipoprotein (LDL) were assessed with an automatic biochemical analyzer (BS-420, Shenzhen, China).

Inflammation-related cytokines (IL-1β, IL-6, TNF-α, IL-10), immunoglobulins (IgA, IgG, IgM), and other indicators such as taurine, cortisol (CORT), insulin-like growth factor-1 (IGF-1), and growth hormone (GH) were measured using enzyme-linked immunosorbent assay (ELISA) kits. All assays followed the manufacturer’s instructions, using ELISA kits from Shanghai Enzyme-linked Biotechnology Co., Ltd., Shanghai, China.

The ferric reducing antioxidant power (FRAP) method was employed to assess total antioxidant capacity (T-AOC). Glutathione peroxidase (GSH-Px) activity was assessed via the DTNB colorimetric method. Superoxide dismutase (SOD) activity was assessed using the xanthine oxidase method. Malondialdehyde (MDA) concentration was measured using the thiobarbituric acid (TBA) method. All assays followed the instructions provided with the kits, which were sourced from Nanjing Jiancheng Bioengineering Institute, Nanjing, China.

#### 2.3.5. Intestinal Tissue Collection and Profile of Intestinal Tissue Transcriptome

Growth performance serves as a core and comprehensive evaluation indicator that systematically reflects the overall nutritional status and heat stress tolerance of lambs, rather than relying on discrete physiological parameters. Accordingly, based on the 63-day growth performance data, the MT group with the optimal comprehensive effects was selected as the representative RPT intervention group for subsequent multi-omics analysis, to further explore the underlying regulatory mechanisms. Jejunal tissue samples (≥2 g) were collected from the same individuals as the microbiome analysis (*n* = 6), promptly frozen in liquid nitrogen, and stored at −80 °C in the laboratory until analysis.

Approximately 1 g of jejunal tissue previously preserved in liquid nitrogen was rapidly ground into fine powder in a pre-chilled, RNase-free mortar with the addition of liquid nitrogen. RNA extraction was performed using the R1200 kit (Solarbio, Beijing, China) following the manufacturer’s instructions. RNA concentration and purity were assessed using a micro-volume spectrophotometer (NanoDrop™ 2000, Thermo Fisher Scientific, Waltham, MA, USA). Samples meeting quality requirements were used for subsequent transcriptomic analysis. Under high-temperature conditions, mRNA was fragmented and served as a template for first-strand cDNA synthesis using reverse transcriptase. Double-stranded cDNA was synthesized using dNTPs and RNase H, followed by end repair, A-tailing, and ligation with Illumina sequencing adapters. The final sequencing library was constructed by PCR amplification and enrichment. The Agilent 2100 Bioanalyzer (Agilent Technologies, Santa Clara, CA, USA) was used to evaluate library quality and concentration, and the qualified libraries were sequenced on the Illumina HiSeq platform.

FastQC v0.12.1 was used for quality control of the raw sequencing data. Adapter sequences and low-quality bases were removed using Trimmomatic v0.40 [41] to obtain high-quality clean reads. Clean reads were aligned to the reference genome via HISAT2 v2.2.2, followed by transcript assembly and quantification using StringTie v3.0.3. After normalization of the gene expression matrix, DESeq2 v1.50.2 facilitated the analysis of differential gene expressions. The Gene Ontology (GO) and Kyoto Encyclopedia of Genes and Genomes (KEGG) databases facilitated the annotation and enrichment of differentially expressed genes (DEGs). Genes related to oxidative stress and inflammatory signaling pathways were further screened. Key gene expression patterns were visualized using volcano plots, and relationship between functions were analyzed and visualized by cnet plot.

To confirm the transcriptome findings, high-quality RNA was converted into cDNA utilizing the RP1105 reverse transcription kit (Solarbio, China). Two upregulated and two downregulated DEGs were selected for validation. Quantitative real-time PCR (qRT-PCR) was conducted with cDNA templates on a QuantStudio™ 1 Real-Time PCR System (Thermo Fisher Scientific, USA). The internal reference gene utilized was β-actin. Each sample was run in triplicate, and the reaction system and cycling parameters followed the SYBR^®^ Green method. Relative gene expression levels were calculated using the 2^−ΔΔCt^ method, with primer sequences for the four genes detailed in Supplementary Table S1.

#### 2.3.6. Gut Content Collection and Profile of Gut Microbiota by Full-Length 16S rRNA Gene Sequencing

On day 64 of the formal trial, six lambs (*n* = 6) were selected from the CN (Control) and MT (Treatment) groups for slaughter. Postmortem jejunal content samples (≥2 g) were immediately frozen in liquid nitrogen and stored at −80 °C until laboratory analysis.

Frozen jejunal content samples were ground thoroughly in liquid nitrogen to obtain a fine powder. Environmental DNA (eDNA) was extracted from the samples using the D2700 DNA extraction kit (Solarbio, China) following the manufacturer’s guidelines. DNA purity, concentration, and integrity were evaluated with a NanoDrop spectrophotometer, while fragment size distribution was examined using agarose gel electrophoresis. The full-length 16S rRNA gene was amplified using specific primers, and the products were quantified with a Qubit fluorometer (Thermo Fisher Scientific, Waltham, MA, USA). Agarose gel electrophoresis was used to confirm the fragment size distribution of the amplification products. The amplified products were then subjected to end repair and dA-tailing, followed by ligation of sequencing adapters. Libraries were purified with magnetic beads, loaded onto an R10.4.1 Flow Cell, and sequenced using a PromethION P48 platform (Oxford Nanopore Technologies, Oxford, UK).

Raw fastq sequences were extracted from the output Fast5 data, followed by barcode identification, adapter trimming, low-quality read filtering, primer recognition, and length filtering. Chimeric sequences were detected and eliminated utilizing Minimap2 v2.30 and yacrd v1.0.0. The remaining sequences were aligned to the reference database using LAST v1650, and only the highest-scoring alignment for each sequence was retained to construct the feature table. Alpha and beta diversity metrics were calculated to assess community richness, diversity, and similarity in QIIME2 v2024.10. Principal coordinate analysis (PCoA) using Bray–Curtis dissimilarity and nonmetric multidimensional scaling (NMDS) with weighted UniFrac distance were performed. These analyses were complemented by nonparametric multivariate analysis of variance (ADONIS) and analysis of similarity (ANOSIM), respectively, using R (www.rproject.org). Linear discriminant analysis effect size (LEfSe) was conducted using QIIME2. Functional Annotation of Prokaryotic Taxa (FAPROTAX) was employed for function annotation analysis [42].

#### 2.3.7. Correlation Analysis and Statistical Analysis

To investigate the relationship among gut microbiota and intestinal epithelial transcriptome, as well as serum parameters, the correlation analysis was performed. To investigate the relationship among gut microbiota and intestinal epithelial transcriptome, as well as serum parameters, the correlation analysis was performed. The study examined the link between differentially expressed intestinal genes and serum parameters to pinpoint specific genes associated with differential pathways. The OmicStudio platform was used to visualize the correlation between gut microbiota, serum parameters, and intestinal genes through correlation network heatmap analysis [43].

The Shapiro–Wilk test was employed to verify the normality of growth performance, rumen fermentation, apparent digestibility, serum physiological and biochemical indicators, as well as microbial and gene abundance data. Subsequently, Analysis of Variance (ANOVA) was employed to compare growth performance, rumen fermentation, apparent digestibility, and serum physiological and biochemical indicators across the four groups. Orthogonal polynomial contrasts were performed to examine linear and quadratic effects of increasing additive levels, and Tukey’s post hoc test was adopted for multiple comparisons when the treatment effect was significant. The comparison of gut microbial and gene abundance was performed via Student’s *t*-test. The statistical analyses utilized SPSS 24.0 software (IBM, Armonk, NY, USA), with significance defined as *p* < 0.05 and a trend toward significance at 0.05 ≤ *p* < 0.10.

## 3. Results

### 3.1. THI in the Sheep Barn and Heat Stress Status of Hu Sheep

**Figure 1:**
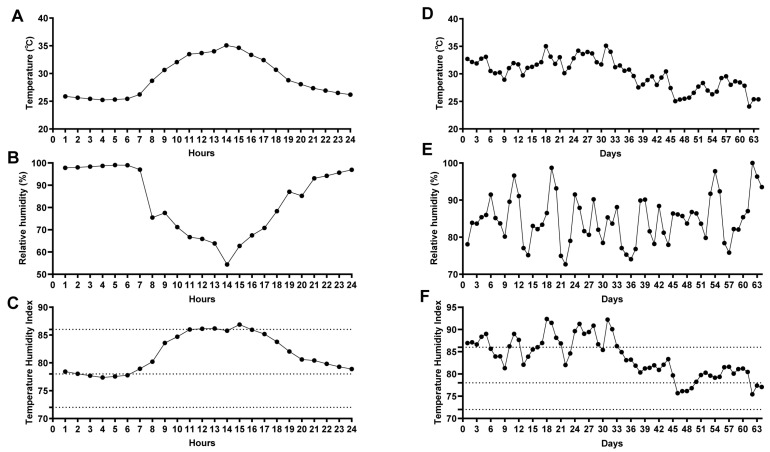
The temperature, relative humidity, and temperature-humidity index (THI) in the sheep barn during experiment. (**A**) Average temperature variation over 24 h in the sheep barn; (**B**) Average relative humidity variation over 24 h in the sheep barn; (**C**) Average THI variation over 24 h in the sheep barn; (**D**) Change in daily average temperature across experimental days in the sheep barn; (**E**) Change in daily average relative humidity across experimental days in the sheep barn; (**F**) Change in daily average THI across experimental days in the sheepfold. The dashed lines represent the THI thresholds of 72 and 78, indicating the starting lines of mild and moderate heat stress, respectively.

Throughout the experimental period, both the 24 h average THI and the daily mean THI in the sheep barn consistently exceeded 72, surpassing the physiological comfort threshold for Hu sheep. This indicates that the experimental animals were continuously exposed to a heat stress environment. Statistical analysis revealed that during the trial, the number of days under mild, moderate, and severe heat stress were 6 days (9.52%), 34 days (53.97%), and 23 days (36.51%), respectively. These results suggest that Hu sheep were predominantly subjected to moderate to severe heat stress throughout the experimental period (Figure 1).

### 3.2. Effects of RPT on Growth Performance of Heat-Stressed Hu Sheep

As shown in Table 2, compared with the 0% control group, dietary supplementation with 0.4% RPT significantly increased FBW, ADG and ADFI while decreasing F/G (*p* < 0.05). No significant differences were observed among the 0%, 0.2% and 0.6% RPT groups (*p* > 0.05). FBW exhibited significant linear (*p* = 0.047) and quadratic (*p* = 0.046) responses to increasing RPT levels. ADG showed a significant linear effect (*p* = 0.038) and a tendency for quadratic response (*p* = 0.073). ADFI had significant linear (*p* = 0.011) and quadratic (*p* < 0.001) effects, and F/G presented a significant quadratic effect (*p* = 0.029) without a significant linear response (*p* = 0.084).

### 3.3. Effects of RPT on Rumen Fermentation of Heat-Stressed Hu Sheep

As shown in Table 3, no significant differences were detected in rumen fermentation parameters across the RPT inclusion groups (*p* > 0.05). Rumen pH tended to increase with increasing dietary RPT levels, showing a significant quadratic response (*p* = 0.015), though the overall difference among groups was not statistically significant (*p* = 0.084). No significant differences were observed in rumen NH_3_-N, MCP, TVFA, propionic acid, valeric acid, or the A/P among treatments (*p* > 0.05). Acetic acid proportion showed a significant linear increase with graded RPT supplementation (*p* = 0.030), while butyric acid proportion exhibited a significant linear decrease (*p* = 0.042).

### 3.4. Effects of RPT on Apparent Digestibility of Nutrients in Heat-Stressed Hu Sheep

As shown in Table 4, no significant differences were observed in DMD, OMD, CPD, or ADFD across treatments (*p* > 0.05). EED tended to differ among groups (*p* = 0.074), with the 0.4% RPT group showing numerically higher EED than the control. NDFD was significantly higher in the 0.2% RPT group than in the control (*p* < 0.05), and exhibited a significant quadratic response to graded RPT inclusion (*p* = 0.011). No significant linear or quadratic effects were detected for other nutrient digestibility parameters (*p* > 0.05).

### 3.5. Effects of RPT on Serum Lipids, Stress, and Growth Hormone Levels in Heat-Stressed Hu Sheep

Table 5 shows that serum HDL concentration was significantly higher in the 0.4% RPT group than in the 0% and 0.2% groups (*p* < 0.05). Serum CORT concentration was significantly lower in the 0.4% RPT group than in the 0% and 0.6% groups (*p* < 0.05), and exhibited a significant quadratic response to graded RPT inclusion (*p* = 0.003). Serum taurine concentrations were significantly elevated in all RPT-supplemented groups compared with the control (*p* < 0.05), with the 0.6% group showing the highest level (*p* < 0.001), and a significant linear increase with increasing RPT levels (*p* < 0.001). No significant differences were observed in serum CHO, TG, LDL, GH, or IGF-1 concentrations across treatments (*p* > 0.05).

### 3.6. Effects of RPT on Serum Antioxidant Levels in Heat-Stressed Hu Sheep

Table 6 shows that serum SOD activity was significantly higher in the 0.2% RPT group than in the control (*p* < 0.05), and exhibited a significant quadratic response to graded RPT inclusion (*p* = 0.015). Serum GSH-Px activity was significantly higher in the 0.2% RPT group than in the 0.4% group (*p* < 0.05). No significant differences were observed in T-AOC across treatments (*p* > 0.05). Serum MDA concentration tended to decrease linearly with increasing RPT levels (*p* = 0.031), although the overall difference among groups was not statistically significant (*p* = 0.108).

### 3.7. Effects of RPT on Serum Immunoglobulin and Cytokine Factors in Heat-Stressed Hu Sheep

According to Table 7, serum IgG concentration decreased linearly with increasing RPT levels (*p* = 0.004), with the 0.6% RPT group showing significantly lower IgG than the control (*p* < 0.05). No significant differences were observed in IgA or IgM across groups (*p* > 0.05). For inflammatory cytokines, serum TNF-α, IL-1β, and IL-6 all decreased linearly with graded RPT supplementation (*p* < 0.001). TNF-α was significantly lower in the 0.4% and 0.6% groups than in the control, with the 0.6% group having the lowest concentration (*p* < 0.05) and a significant quadratic response (*p* = 0.023). IL-1β was significantly reduced in the 0.4% and 0.6% groups relative to the control, and the 0.6% group was also lower than the 0.2% group (*p* < 0.05). IL-6 was significantly lower in all RPT-supplemented groups compared to the control, with further stepwise reductions across the 0.2%, 0.4%, and 0.6% levels (*p* < 0.05). Conversely, the anti-inflammatory cytokine IL-10 increased linearly with increasing RPT inclusion (*p* < 0.001), being significantly higher in the 0.4% and 0.6% groups than in the control, and highest in the 0.6% group (*p* < 0.05).

### 3.8. Effects of RPT on the Jejunal Transcriptome in Heat-Stressed Hu Sheep: Focus on Inflammatory Responses

**Figure 2:**
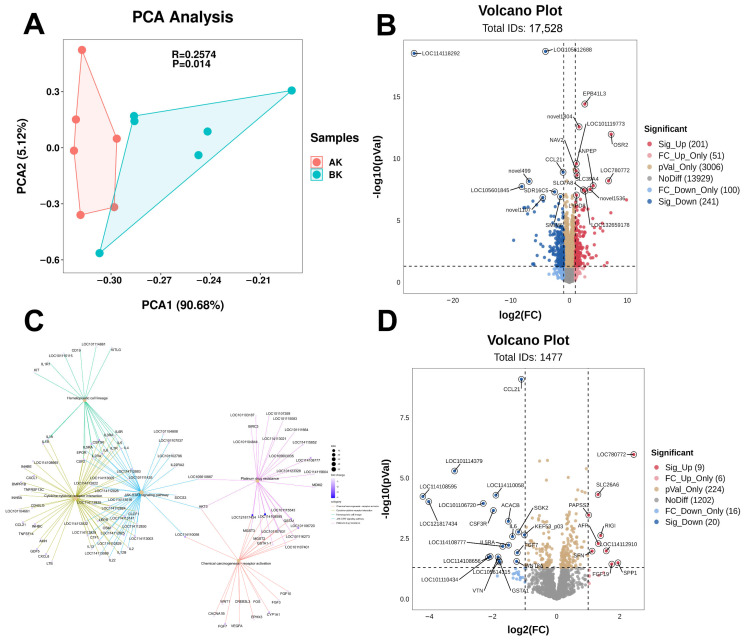
Transcriptomic profile of intestinal epithelium in heat-stressed Hu sheep fed diets supplemented with rumen-protected taurine (RPT). (**A**) Principal Component Analysis (PCA) of samples from two groups, AK represents the control group fed the basal diet without RPT; BK represents the group offered the basal diet supplemented with 0.4% RPT. (**B**) Volcano plot showing differentially expressed genes between the two groups; (**C**) gene network diagram of inflammation-related pathways; (**D**) volcano plot identifying inflammation-related differentially expressed genes between the control and MT groups.

A total of 516,607,144 high-quality sequences (clean reads) were obtained from 12 individuals, with an average of 43,050,595 high-quality sequences per individual (ranging from 42,974,524 to 43,174,226). The average alignment rate of high-quality reads to the reference genome was 98.08%, indicating the reliability of the sequencing results and the accuracy of the reference genome (Table S2). A total of 17,528 genes were identified across 12 sheep samples. Principal component analysis (PCA) based on relative gene expression revealed a significant difference in gene distribution between the two groups (Figure 2A). This difference was statistically validated by ANOSIM analysis (R = 0.26, *p* = 0.014).

DEGs were screened with the thresholds of ∣*log*_2_ FC∣ > 1 and adjusted *p* < 0.05. The Benjamini–Hochberg method was applied for multiple testing correction to control the false discovery rate. A total of 442 DEGs were identified, including 201 upregulated and 241 downregulated genes (Figure 2B). Gene Ontology (GO) enrichment analysis of the DEGs revealed significant enrichment in biological processes related to immunity, such as immune response, immune system process, and defense response. DEGs were primarily associated with cellular components such as intrinsic membrane components, extracellular regions and spaces, membranes, and the MHC class I protein complex. The DEGs were primarily associated with molecular functions such as transporter activity, transmembrane transporter activity, oxidoreductase activity, and 2′-5′-oligoadenylate synthetase activity (Supplementary Figure S1A). KEGG pathway enrichment analysis further demonstrated that, aside from Metabolism, the majority of DEGs were enriched in the Human Diseases category, primarily associated with viral infections. In this study, within the Cellular Processes category, genes were primarily enriched in the Phagosome pathway. Meanwhile, in the Environmental Information Processing category, the enrichment was mainly associated with ABC transporters (Supplementary Figure S1B).

Subsequently, immune- and inflammation-related pathways were reanalyzed, resulting in a pathway enrichment map as shown in Supplementary Figure S2. Gene-pathway network analysis identified crucial pathways such as JAK-STAT signaling, cytokine-cytokine receptor interaction, hematopoietic cell lineage, platinum drug resistance, and chemical carcinogenesis via receptor activation (Figure 2C). Among these, the JAK-STAT signaling pathway appeared to be a prominent node within the regulatory network together with the cytokine-cytokine receptor interaction and hematopoietic cell lineage pathways (Supplementary Figure S3). Then, a total of 1477 genes associated with these key pathways were selected for subsequent analysis, and 29 DEGs were identified. Notably, several inflammation-related genes—such as *CCL21*, *CSF3R*, *IL6*, and *IL5RA*—were significantly downregulated in the MT group (Figure 2D). To validate the transcriptomic findings, two upregulated genes (*LOC780772* and *SLC26A6*) and two downregulated genes (*CCL21* and *IL6*) were selected for qRT-PCR analysis, which confirmed expression trends consistent with the RNA-seq data (Supplementary Figure S4).

### 3.9. Effects of RPT on Gut Microbiota in Heat-Stressed Hu Sheep

**Figure 3:**
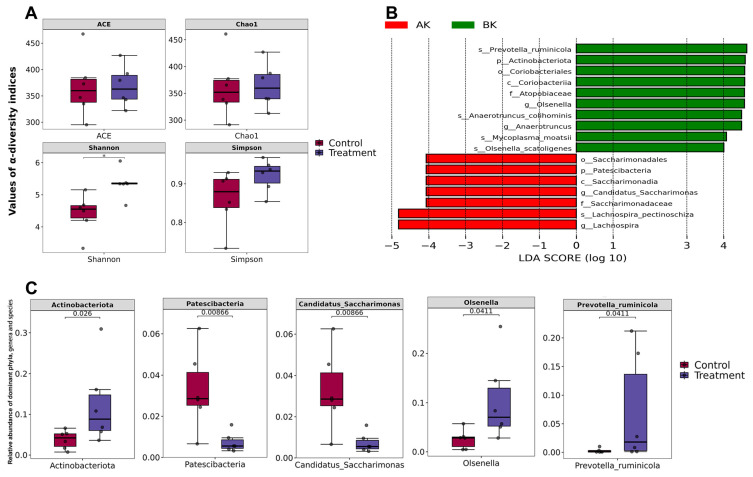
Profile of gut microbial α-diversity, β-diversity, and community composition in heat-stressed Hu sheep fed diets supplemented with RPT. (**A**) Comparison of gut microbial α-diversity between the CON group (0% RPT) and the treatment group (0.4% RPT), * means *p* < 0.05; (**B**) Linear discriminant analysis effect size (LEfSe, threshold = 4) of gut microbial composition between the AK (0% RPT) and the BK (0.4% RPT) groups; (**C**) comparison of intestinal microbiota at phylum, genus and species level.

After filtering, 826,388 valid sequences were obtained from 12 samples, averaging 68,866 sequences per sample with a mean length of 1447 bp (Supplementary Table S3). The rarefaction curves demonstrated that the sequencing depth had plateaued, indicating that additional sequencing would not increase species diversity (Supplementary Figure S5). Among the α-diversity indices, only the Shannon index showed a significant difference, with the 0.4% supplementation level exhibiting a higher value than the CON (0% supplementation) (*p* < 0.05), while the other indices remained similar (Figure 3A; Supplementary Table S3).

PCoA based on weighted UniFrac distances clearly showed no obvious overlap between individuals from the two groups, indicating substantial differences between them (Supplementary Figure S6A). ADONIS analysis of weighted UniFrac distances revealed a significant group difference (R = 0.31, *p* = 0.005). NMDS using Bray–Curtis distances showed significant overlap in sample distribution between the groups (Supplementary Figure S6B). Despite the redundancy, ANOSIM of Bray–Curtis distance matrices revealed a significant difference between the groups (R = 0.57, *p* = 0.003).

**Figure 4:**
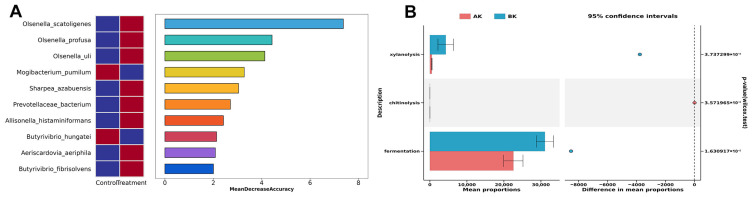
Random forest analysis and predicted functions of intestinal microbiota in heat-stressed Hu sheep between rumen-protected taurine (RPT) supplementation and control group. (**A**) random forest analysis of intestinal microbiota identifying top contributing biomarkers at species level in each group, the heatmap on the left shows the relative abundance of microbiota; red indicates higher abundance, and blue indicates lower abundance; (**B**) comparison of differential functions of intestinal microbiota.

Using LEfSe with a threshold of 4, phylogenetic analysis indicated that the 0.4% supplementation level showed a significant increase in 10 bacterial taxa and a decrease in 7 taxa, spanning 2 phyla, 2 classes, 2 orders, 2 families, 4 genera, and 5 species (Figure 3B). Analysis of dominant taxa revealed that within the top 10 abundant phyla (Supplementary Figure S7), the 0.4% supplementation level exhibited a significantly higher relative abundance of Actinobacteriota and a significantly lower abundance of Patescibacteria (Figure 3C). At the genus level, the MT group exhibited a significantly lower relative abundance of Candidatus Saccharimonas and a significantly higher abundance of *Olsenella* (Figure 3C and Figure S8). At the species level, Prevotella ruminicola abundance was significantly higher in the 0.4% supplemental group (Figure 3C and Figure S9). Random forest analysis at the genus level revealed that *Olsenella* was the dominant genus contributing most to the differences in the 0.4% supplemental group (Supplementary Figure S10). Similarly, random forest analysis at the species level identified three dominant species within the genus *Olsenella*—*Olsenella scatoligenes*, *Olsenella profusa*, and *Olsenella uli*—as contributing most to the differences in the 0.4% supplemental group (Figure 4A). Using FAPROTAX for functional prediction of the gut microbiota revealed 77 functional clusters. Notably, only three exhibited significant differences: xylanolysis and fermentation functions were elevated in the taurine-supplemented group, while chitinolysis was more pronounced in the control group (Figure 4B).

### 3.10. Correlation Analysis Among Gut Microbiota and Intestinal Epithelial Transcriptome, as Well as Serum Parameters

**Figure 5:**
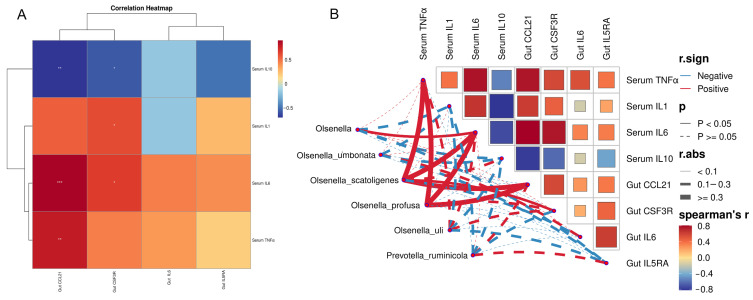
Correlation analysis among three indicators in heat-stressed Hu sheep: intestinal microbiota biomarker genera and species, serum inflammatory factors, and intestinal inflammation-related differentially expressed genes (DEGs). (**A**) correlation heatmap analysis between serum inflammatory factors and intestinal inflammation-related DEGS, * means *p* < 0.05, ** means *p* < 0.01, *** means *p* < 0.001; (**B**) network heatmap analysis of correlations among intestinal microbiota biomarker genera and species, serum inflammatory factors, and intestinal inflammation-related DEGs.

First, Pearson correlation analysis was performed between serum inflammatory markers (TNFα, IL1, IL6, IL10) and inflammation-related differentially expressed genes in the intestinal tissue (*CCL21*, *CSF3R*, *IL6*, *IL5RA*). The study found that the *CCL21* gene exhibited a significant positive correlation with serum TNFα and IL6, and a significant negative correlation with IL10. Similarly, the *CSF3R* gene was significantly positively correlated with serum IL1 and IL6, while showing a significant negative correlation with IL10 (Figure 5A). Correlation network analysis revealed significant positive associations between the genus *Olsenella* and serum IL6. Specifically, *Olsenella scatoligenes* showed significant positive correlations with serum TNFα and IL6, along with intestinal *CCL21* and *CSF3R* genes. Similarly, *Olsenella profusa* was significantly positively correlated with serum TNFα and IL6, as well as the intestinal *CCL21* gene (Figure 5B).

## 4. Discussion

High temperatures in summer usually lead to a significant increase in serum cortisol levels of Hu sheep, accompanied by elevated body temperature and accelerated respiratory rate. Moreover, the abnormal degree of these heat stress-related indicators is strongly correlated with the THI [44,45]. During the experimental period, both the 24 h average and daily mean THI in the sheep barn consistently exceeded 72, indicating prolonged exposure of Hu sheep to heat stress. Over 90% of the trial days experienced moderate to severe heat stress, offering a strong environmental framework to assess the impact of RPT supplementation on physiological, transcriptomic, immune, and microbial responses. Dietary RPT did not significantly impact rumen fermentation parameters in heat-stressed Hu sheep. This finding aligns with prior studies on rumen-protected taurine [20,23] and contrasts with findings that unprotected taurine significantly affects rumen fermentation parameters [46,47,48]. We speculate that RPT avoids ruminal degradation, and the rumen maintains dynamic functional homeostasis. In addition, rumen samples were collected only at a single time point under fasting conditions, which together resulted in no significant differences in rumen fermentation parameters among treatments.

RPT supplementation differentially affected the growth performance of heat-stressed Hu sheep. Moderate RPT inclusion at 0.4% effectively improved growth and feed efficiency, confirming a favorable response to appropriate dosage. The graded rise in FBW, ADG and ADFI along increasing RPT levels reflected a clear dose-dependent growth-promoting effect. Meanwhile, the curvilinear responses of growth and feed efficiency indices implied that the beneficial effects of RPT were not linearly cumulative but presented a typical parabolic trend, with the optimal efficacy achieved at the moderate 0.4% addition and weakened at higher inclusion levels. The absence of noticeable growth benefits in the low and high RPT groups further validated that an optimal dosage window exists for RPT to maximize the production performance of heat-stressed sheep. Previous studies in heat-stressed ruminants reported that folic acid combined with taurine improved ADG, though the effect of RPT alone was not assessed [49]. Studies indicate that RPT enhances ADG in beef cattle under non-heat-stress conditions [23,24], while rumen-protected glucose (RPG) with taurine (TAU) mitigates transport stress-related weight loss in yaks without exerting adverse effects [22]. The current results are consistent with previous findings, confirming that RPT supplementation improves growth performance in ruminants.

The level of RPT supplementation had distinct effects on nutrient digestibility in heat-stressed Hu sheep. Specifically, EED was numerically elevated at the 0.4% RPT level, and NDFD was significantly higher at the 0.2% supplementation level relative to the control group. Among all nutrient digestibility indices, only NDFD presented a significant quadratic response to graded RPT inclusion, implying a curvilinear dose effect of RPT on fiber digestibility with an optimal beneficial dosage. In contrast to findings in beef cattle, where RPT showed no effect on digestibility [23], unprotected taurine was reported to improve NDF digestibility [44,48]. The observed discrepancy might be attributed to heat stress in this study, where RPT potentially improved digestive efficiency by enhancing nutrient absorption in the intestines. Further studies on RPT and its underlying mechanisms are needed to fully and accurately elucidate its effects on the apparent nutrient digestibility of ruminants.

Serum taurine concentrations were significantly elevated in all RPT-supplemented groups compared to the CON group and exhibited linear increase with rising RPT inclusion levels, with the 0.6% supplementation level showing the greatest elevation, aligning with prior studies [23,24]. In contrast, unprotected taurine did not increase blood taurine concentrations in beef cattle [48], indicating that rumen protection is essential for effectively elevating blood taurine levels. RPT supplementation also altered serum lipid and stress hormone profiles. The 0.4% RPT level increased HDL and lowered cortisol relative to corresponding groups. The significant quadratic response of cortisol to increasing RPT doses indicates a curvilinear regulatory effect on heat stress, with the moderate 0.4% dose presenting the optimal efficacy. Collectively, these findings suggest that appropriate RPT inclusion can improve lipid metabolism and suppress HPA axis overactivation under heat stress. Studies in yaks [20] and beef cattle [24] reported no effect of RPT on serum cortisol or lipid metabolism, likely because these animals were in non-stressed conditions. This study found that cortisol levels in heat-stressed Hu sheep increased but normalized with RPT supplementation, consistent with findings in transport-stressed yaks [22]. Comparable findings have been observed in non-ruminants, with taurine enhancing lipid catabolism [50]. Antioxidant results indicated that the 0.2% RPT group had significantly higher SOD and GSH-Px activities, implying 0.2% dietary RPT effectively enhanced enzymatic antioxidant capacity. Taurine’s ability to boost antioxidant capacity is well-documented, with both rumen-protected [21,22,49] and unprotected forms [27,51] shown to selectively enhance certain blood antioxidant parameters in animals.

RPT significantly influenced serum immunoglobulin and inflammatory cytokine levels in heat-stressed Hu sheep, aligning with earlier research on taurine supplementation in ruminants [22,49]. The gradual decline in serum IgG along the RPT dosage gradient reflected a clear linear dose-dependent trend, with the highest inclusion level showing a notable reduction relative to the control. This implies that excessive RPT supplementation may exert a certain inhibitory effect on humoral immune responses, which is partially supported by previous yak studies reporting superior immune regulation under moderate supplementation dosage [20]. Graded RPT supplementation linearly decreased serum TNF-α, IL-1β and IL-6 levels, implying that RPT could suppress pro-inflammatory overproduction and alleviate heat stress-induced inflammation [52]. The curvilinear response of TNF-α to RPT dosage further indicated a non-linear regulatory pattern in inflammatory modulation. The stepwise decline of IL-6 with increasing RPT doses also reflected a consistent anti-inflammatory effect across all supplementation levels [52]. Meanwhile, the anti-inflammatory cytokine IL-10 rose linearly as RPT inclusion increased, with higher doses presenting stronger upregulation. The results corroborate earlier studies indicating that taurine mitigates inflammation by modulating signaling pathways, reducing pro-inflammatory cytokines like TNF-α, IL-1β, and IL-6, and enhancing IL-10 levels [53]. Under heat stress, dietary supplementation with 0.6% RPT exhibited the strongest anti-inflammatory effect but reduced IgG levels, unlike studies in non-heat-stress conditions where immunoglobulin levels remained unchanged [20,49]. Such discrepancy may be attributed to elevated immune sensitivity under heat stress, where high-dose RPT exerts strong anti-inflammatory effects while partially restraining immunological activity.

Transcriptomic analysis of the small intestine in Hu sheep revealed that RPT treatment exerted significant transcriptional effects under heat stress, suggesting a key role of RPT in modulating intestinal function. Although no prior studies have specifically examined the impact of taurine on the intestinal transcriptome of ruminants, Chen et al. [21] reported that combined supplementation of RPG and RPT altered hepatic gene expression patterns in yaks using qRT-PCR. Similarly, Zhang et al. [24] found that RPT modified the blood transcriptome profile in beef cattle, while studies in pigs demonstrated that taurine supplementation significantly changed gene expression in intestinal mucosa [52]. GO enrichment analysis of DEGs indicated that RPT may act through pathways related to membrane-associated immune recognition, substance transport, and antiviral responses. KEGG pathway analysis further revealed that DEGs were highly enriched in viral infection, phagosome, and ABC transporter pathways, aligning with previous findings on taurine’s role in regulating immune recognition, antiviral defense, and transmembrane transport [54,55,56]. These results suggest that RPT may alleviate heat stress–induced immune dysregulation via coordinated modulation of multiple signaling pathways. A stable regulatory network was identified through further analysis of immune and inflammation-related pathways, with the JAK-STAT signaling pathway being one of the notably enriched pathways, alongside cytokine-cytokine receptor interaction and hematopoietic cell lineage. These pathways are crucial for inflammation, cell differentiation, and immune signal transduction [57,58]. The study identified 29 DEGs linked to these pathways, with notable downregulation of inflammation-related genes such as *CCL21*, *CSF3R*, *IL6*, and *IL5RA* in the RPT-treated group, suggesting that RPT may reduce inflammatory gene expression to alleviate heat stress-induced inflammation. Chen et al. (2025) found that RPT supplementation notably reduced the expression of inflammatory genes, including TLR4, IL-8, IL-1β, and TNF-α, in the livers of yaks. [21]. Correlation analysis between these DEGs and serum inflammatory cytokine levels (Figure 5A) showed that the four downregulated genes were generally positively correlated with pro-inflammatory cytokines (except IL6 with serum IL-1), and negatively correlated with anti-inflammatory cytokines (IL10), suggesting consistency between intestinal and systemic anti-inflammatory effects of RPT. Taurine’s anti-inflammatory effects have been suggested to involve modulation of pathways such as NF-κB and JAK-STAT in other studies, and our data are consistent with a potential role for these pathways under heat stress [59,60]. This study found that RPT significantly regulates inflammation-related genes under heat stress, with the strongest effects observed in the medium-dose group, aligning with earlier results under transport stress or pathological conditions [22].

This study utilized full-length 16S rRNA gene sequencing to systematically evaluate the effects of RPT on the gut microbiota’s structure and function in heat-stressed Hu sheep. β-diversity analysis confirmed the overall impact of RPT on gut microbial ecology, while α-diversity analysis revealed that RPT significantly increased Shannon diversity, indicating improved microbial community structure. Previous studies have similarly shown that both RPT and unprotected taurine can enhance rumen microbial diversity [46,49]. Taxonomic analysis showed that RPT raised the relative abundance of Actinobacteriota phylum while reducing that of Patescibacteria, without significantly affecting the dominant phyla Firmicutes and Bacteroidetes. This aligns with earlier findings that RPT has limited effects on rumen microbiota [49], whereas unprotected taurine significantly alters Firmicutes abundance [46,48]. At the genus level, *Olsenella* was notably enriched, whereas *Candidatus Saccharimonas*, a potential pathogenic taxon linked to poor health [61], was diminished, indicating taurine’s positive influence on microbial composition. *Prevotella ruminicola*, a crucial fibrolytic and fermentative bacterium in the ruminant gastrointestinal tract [62], was significantly elevated in the 0.4% RPT group. Random forest analysis further identified *Olsenella* and its three dominant species as major contributors to the microbial differences in the RPT-treated group, suggesting that *Olsenella* species may be associated with host biological responses following RPT supplementation, though their functional role requires further investigation. Consistent with current findings, prior research indicates *Olsenella*’s significant role in host immune regulation, short-chain fatty acid metabolism, and maintaining mucosal barrier integrity [63,64]. Functional prediction revealed that RPT significantly enhanced microbial xylanolysis and fermentation functions, suggesting that RPT may promote fiber degradation and fermentation, thereby improving nutrient utilization and alleviating heat stress–induced digestive suppression, which are consistent with the increased abundance of *Prevotella ruminicola* and *Olsenella* [62,64]. Further correlation analysis demonstrated that *Olsenella* and its three dominant species were positively associated with serum IL-6 and TNF-α concentrations, as well as intestinal expression of the *CCL21* and *CSF3R* genes, which suggest that *Olsenella* may participate in RPT’s anti-inflammatory effects by modulating inflammatory gene expression. Research indicates that gut microbiota can affect host inflammatory responses by modulating immune pathways like JAK-STAT and NF-κB [65,66], with *Olsenella* playing a role in mucosal immune regulation and inflammation management [62]. This study is the first to show a significant link between *Olsenella* and both inflammatory cytokines and intestinal immune gene expression in heat-stressed Hu sheep, suggesting a mechanism by which RPT enhances intestinal immune balance through targeted microbial modulation.

## 5. Conclusions

This study systematically assessed the physiological regulatory effects of RPT on heat-stressed Hu sheep, demonstrating its efficacy in mitigating the adverse impacts of heat stress across various dimensions. The 0.4% RPT supplementation significantly improved growth performance and stress hormone levels, while enhancing antioxidant activity and modulating immune and inflammatory markers. Transcriptomic analysis revealed that RPT downregulated pro-inflammatory gene expression by regulating pathways such as JAK-STAT, and microbiome profiling showed significant enrichment of *Olsenella* in the RPT-treated group, suggesting a potential link between these microbes and the anti-inflammatory effects of RPT. Overall, RPT supplementation, especially at 0.4% level, showed strong potential for mitigating heat stress, improving intestinal function, and maintaining immune homeostasis, thereby supporting its application as a functional feed additive.

Future studies should employ cell-based experiments to validate the specific mechanisms by which RPT regulates inflammatory signaling pathways such as JAK-STAT pathway and identify its molecular targets in immune modulation. Additionally, fecal microbiota isolation and culture techniques could be used to functionally characterize representative strains of the genus *Olsenella*.

## Figures and Tables

**Table 1:** Nutrient composition and ingredients of the basal diet (dry matter basis).

Ingredients	Contents (%)
Wheat straw	18.82
Peanut vine	21.25
Corn grain	29.59
Wheat bran	10.38
Soybean meal	15.35
Calcium hydrogen phosphate	0.84
Sodium bicarbonate	0.27
Salt	0.50
Premix ^1^	3.00
Total	100
Nutrient levels ^2^	
CP	14.20
EE	3.28
NDF	32.86
ADF	14.68
Calcium	0.64
Phosphorus	0.35
ME (MJ/kg)	9.70

^1^ The premix provided the following per kg of diet: VA 10,000 IU, VD 2000 IU, VE 80 IU, Cu 25 mg, Fe 60 mg, Mn 30 mg, Zn 60 mg. ^2^ Metabolic energy is calculated as the sum of ME of each feedstuff by its proportion in diets, other nutrient levels are measured values.

**Table 2:** Effects of rumen-protected taurine (RPT) supplementation on growth performance of Hu sheep under heat stress.

Growth Performance Indices	Dietary RPT Inclusion Levels (%)				SEM	*p* Value	*p* Value	
	0	0.2	0.4	0.6			Linear	Quadratic
IBW (kg)	26.81	27.30	26.76	26.87	0.42	0.970	0.934	0.826
FBW (kg)	41.09 ^b^	43.46 ^ab^	43.96 ^a^	43.15 ^ab^	0.83	0.032	0.047	0.046
ADG (kg/d)	0.23 ^b^	0.26 ^ab^	0.28 ^a^	0.26 ^ab^	0.01	0.033	0.038	0.073
ADFI (kg/d)	1.32 ^b^	1.37 ^ab^	1.40 ^a^	1.36 ^ab^	0.04	0.004	0.011	<0.001
F/G	5.88 ^a^	5.31 ^ab^	5.09 ^b^	5.39 ^ab^	0.15	0.049	0.084	0.029

Note: In the same row, values with no letter superscripts mean no significant difference (*p* > 0.05), while with different letter superscripts mean significant difference (*p* < 0.05).

**Table 3:** Effects of RPT supplementation on rumen fermentation of Hu sheep under heat stress.

Rumen Fermentation Parameters	Dietary RPT Inclusion Levels (%)				SEM	*p* Value	*p* Value	
	0	0.2	0.4	0.6			Linear	Quadratic
pH	6.87	7.00	7.05	6.91	0.03	0.084	0.481	0.015
NH_3_-N (mg/dL)	25.01	25.42	26.54	26.92	1.45	0.965	0.617	0.995
MCP (mg/dL)	93.56	93.23	88.56	87.92	2.29	0.756	0.318	0.971
TVFA (mmol/L)	43.27	40.02	43.16	41.07	1.34	0.676	0.528	0.957
Acetic acid (%)	54.42	55.95	58.07	57.19	0.55	0.355	0.030	0.516
Propionic acid (%)	29.52	28.16	27.75	28.48	0.84	0.905	0.656	0.556
Butyric acid (%)	10.56	10.78	9.38	8.95	0.46	0.173	0.042	0.478
Valeric acid (%)	1.90	2.20	2.10	1.93	0.17	0.353	0.973	0.089
A/P	1.88	2.14	2.19	2.07	0.09	0.650	0.440	0.316

**Table 4:** Effects of RPT supplementation on apparent digestibility of nutrients in Hu sheep under heat stress.

Apparent Digestibility Parameters (%)	Dietary RPT Inclusion Levels (%)				SEM	*p* Value	*p* Value	
	0	0.2	0.4	0.6			Linear	Quadratic
DMD	77.65	77.48	79.06	77.85	0.84	0.583	0.304	0.392
OMD	81.39	83.59	84.22	82.31	0.95	0.752	0.709	0.320
CPD	72.98	76.31	76.43	72.94	0.89	0.314	0.999	0.067
EED	73.97 ^a^	76.25 ^ab^	78.95 ^a^	76.11 ^ab^	0.71	0.074	0.106	0.052
NDFD	55.28 ^b^	62.82 ^a^	58.99 ^ab^	56.08 ^ab^	1.09	0.040	0.858	0.011
ADFD	46.53	50.08	52.33	49.11	0.95	0.186	0.225	0.077

Note: In the same row, values with no letter superscripts mean no significant difference (*p* > 0.05), while with different letter superscripts mean significant difference (*p* < 0.05).

**Table 5:** Effects of RPT supplementation on serum taurine, lipids, stress, and growth hormone levels in Hu sheep under heat stress.

Parameters	Dietary RPT Inclusion Levels (%)				SEM	*p* Value	*p* Value	
	0	0.2	0.4	0.6			Linear	Quadratic
CHO (mmol/L)	1.46	1.36	1.49	1.42	0.04	0.696	0.966	0.869
TG (mmol/L)	0.25	0.22	0.24	0.25	0.01	0.751	0.786	0.439
HDL (mmol/L)	0.79 ^b^	0.76 ^b^	0.89 ^a^	0.81 ^ab^	0.02	0.041	0.198	0.480
LDL (mmol/L)	0.43	0.44	0.47	0.44	0.02	0.776	0.573	0.536
CORT (ng/mL)	9.06 ^a^	7.81 ^ab^	6.36 ^b^	9.26 ^a^	0.36	0.010	0.772	0.003
GH (ng/mL)	14.32	14.99	15.40	15.51	0.31	0.538	0.167	0.661
IGF-1 (ng/mL)	220.42	232.31	249.89	251.68	6.75	0.310	0.072	0.708
Taurine (pg/mL)	39.73 ^c^	46.12 ^b^	45.92 ^b^	53.86 ^a^	1.14	<0.001	<0.001	0.602

Note: In the same row, values with no letter superscripts mean no significant difference (*p* > 0.05), while with different letter superscripts mean significant difference (*p* < 0.05).

**Table 6:** Effects of RPT supplementation on serum antioxidant parameters in Hu sheep under heat stress.

Antioxidant Parameters	Dietary RPT Inclusion Levels (%)				SEM	*p* Value	*p* Value	
	0	0.2	0.4	0.6			Linear	Quadratic
SOD (U/mL)	11.21 ^b^	13.53 ^a^	12.09 ^ab^	11.39 ^ab^	0.32	0.035	0.734	0.015
GSH-Px (U/mL)	197.18 ^ab^	229.36 ^a^	192.27 ^b^	201.27 ^ab^	4.80	0.020	0.519	0.183
T-AOC (mmol/L)	0.26	0.27	0.29	0.28	0.01	0.213	0.105	0.268
MDA (nmol/mL)	2.72	2.58	2.60	2.13	0.09	0.108	0.031	0.362

Note: In the same row, values with no letter superscripts mean no significant difference (*p* > 0.05), while with different letter superscripts mean significant difference (*p* < 0.05).

**Table 7:** Effects of RPT supplementation on serum immunoglobulin and cytokine factors in Hu sheep under heat stress.

Immunoglobulin and Cytokine Factors	Dietary RPT Inclusion Levels (%)				SEM	*p* Value	*p* Value	
	0	0.2	0.4	0.6			Linear	Quadratic
IgA (g/L)	1.04	1.23	1.1	0.94	0.06	0.483	0.488	0.194
IgG (g/L)	16.29 ^a^	15.47	14.09	11.35 ^b^	0.64	0.027	0.004	0.409
IgM (g/L)	0.62	0.58	0.62	0.55	0.02	0.621	0.402	0.792
		
TNF-α (pg/mL)	57.93 ^a^	53.73 ^ab^	49.83 ^b^	37.17 ^c^	1.62	<0.001	<0.001	0.023
IL-1β (pg/mL)	26.38 ^a^	24.15 ^ab^	21.56 ^bc^	19.44 ^c^	0.59	<0.001	<0.001	0.942
IL-6 (pg/mL)	184.52 ^a^	170.94 ^b^	162.02 ^c^	148.44 ^d^	2.57	<0.001	<0.001	0.999
IL-10 (pg/mL)	9.87 ^c^	11.16 ^bc^	12.41 ^b^	14.53 ^a^	0.37	<0.001	<0.001	0.328

Note: In the same row, values with no letter superscripts mean no significant difference (*p* > 0.05), while with different letter superscripts mean significant difference (*p* < 0.05).

## Data Availability

The raw sequence data of microbiome (GSA: CRA030048) and transcriptome (GSA: CRA030066) reported in this paper have been deposited in the Genome Sequence Archive in National Genomics Data Center, China National Center for Bioinformation/Beijing Institute of Genomics, Chinese Academy of Sciences that are publicly accessible at https://ngdc.cncb.ac.cn/gsa (accessed on 15 September 2025).

## Supplementary Materials

The following supporting information can be downloaded at: https://www.mdpi.com/article/10.3390/ani16101445/s1, Figure S1: Transcriptomic profile of intestinal epithelium in heat-stressed Hu sheep fed diets supplemented with rumen-protected taurine (RPT). A, Gene Ontology (GO) enrichment analysis of differentially expressed genes; B, Kyoto Encyclopedia of Genes and Genomes (KEGG) pathway enrichment analysis of differentially expressed genes; Figure S2: Dotplot of KEGG enrichment analysis of intestinal transcriptome in heat-stressed Hu sheep fed diets supplemented with rumen-protected taurine (RPT); Figure S3: Kegg pathway network diagram focusing on inflammation-related pathways of intestinal transcriptome in heat-stressed Hu sheep fed diets supplemented with rumen-protected taurine (RPT); Figure S4: Boxplot of relative expression levels validating two upregulated and downregulated differentially expressed genes from the transcriptome using real-time quantitative PCR, AK represents the control group with 0% RPT supplementation, and BK represents the group supplemented with 0.4% RPT, relative expression levels in the control group are all 1; Figure S5: The rarefaction curve plot of microbial community in 12 samples of jejunal contents of Hu sheep. A1–A6 represent the six samples from the control group with 0% RPT supplementation, while B1–B6 represent the six samples from the group supplemented with 0.4% RPT; Figure S6: Profile of gut microbial β-diversity in heat-stressed Hu sheep fed diets supplemented with RPT. A, Principal coordinates analysis (PCoA) and B, Non-metric multidimensional scaling (NMDS) based on gut microbial β-diversity between the 0% RPT supplementation group (AK) and the 0.4% RPT supplementation group (BK); Figure S7: Comparison of intestinal microbiota at phylum level in heat-stressed Hu sheep. AK represents the control group with 0% RPT supplementation, and BK represents the group supplemented with 0.4% RPT; Figure S8: Comparison of intestinal microbiota at genus level in heat-stressed Hu sheep. AK represents the control group with 0% RPT supplementation, and BK represents the group supplemented with 0.4% RPT; Figure S9: Comparison of intestinal microbiota at species level in heat-stressed Hu sheep. AK represents the control group with 0% RPT supplementation, and BK represents the group supplemented with 0.4% RPT; Figure S10: Random forest analysis of intestinal microbiota at genus level in heat-stressed Hu sheep; Table S1: Primers for quantitative real-time PCR (*Ovis aries*); Table S2: Clean read counts and reference genome alignment rates in the liver transcriptomes of 12 Hu sheep individuals; Table S3. Microbial sequences and α-diversity indices in jejunal contents of Hu sheep.

## Abbreviations

The following abbreviations are used in this manuscript:

RPT	rumen-protected taurine
CON	Control
LT	Low Level RPT
MT	Moderate Level RPT
HT	High Level RPT
TMR	total mixed ration
THI	temperature-humidity index
DM	dry matter
CP	Crude Protein
EE	Ether Extract
NDF	Neutral Detergent Fiber
ADF	Acid Detergent Fiber
ME	Metabolic energy
IBW	initial body weight
FBW	final body weight
ADG	average daily gain
ADFI	average daily feed intake
F/G	feed-to-gain ratio
NH_3_-N	ammonia nitrogen
MCP	microbial protein
VFA	volatile fatty acids
AIA	Acid-insoluble ash
CHO	cholesterol
TG	triglycerides
HDL	high-density lipoprotein
LDL	low-density lipoprotein
T-AOC	Total antioxidant capacity
GSH-Px	Glutathione peroxidase
SOD	Superoxide dismutase
MDA	Malondialdehyde
DEGs	Differentially expressed genes
PCoA	Principal coordinate analysis
NMDS	nonmetric multidimensional scaling
LEfSe	Linear discriminant analysis effect size
