# Supplementary material for: Rumen-Protected Taurine Alleviates Heat Stress Injury in Hu Sheep by Regulating Inflammatory Response, Gut Microbiota and Transcriptome
Source: Animals (Basel). 2026 May 8;16(10):1445. doi: 10.3390/ani16101445 (PMC13203344; doi:10.3390/ani16101445)
Supplement: Supplementary file 1 [file animals-16-01445-s001.zip › animals-4277087-supplementary.pdf]

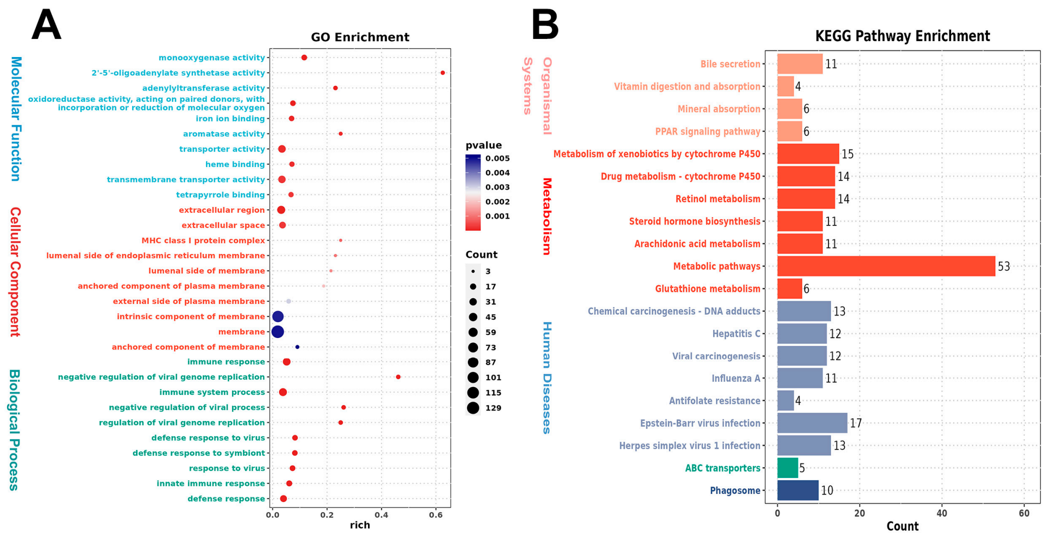

Figure S1. Transcriptomic profile of intestinal epithelium in heat-stressed Hu sheep fed diets supplemented with rumen-protected taurine (RPT). A, Gene Ontology (GO) enrichment analysis of differentially expressed genes; B, Kyoto Encyclopedia of Genes and Genomes (KEGG) pathway enrichment analysis of differentially expressed genes.

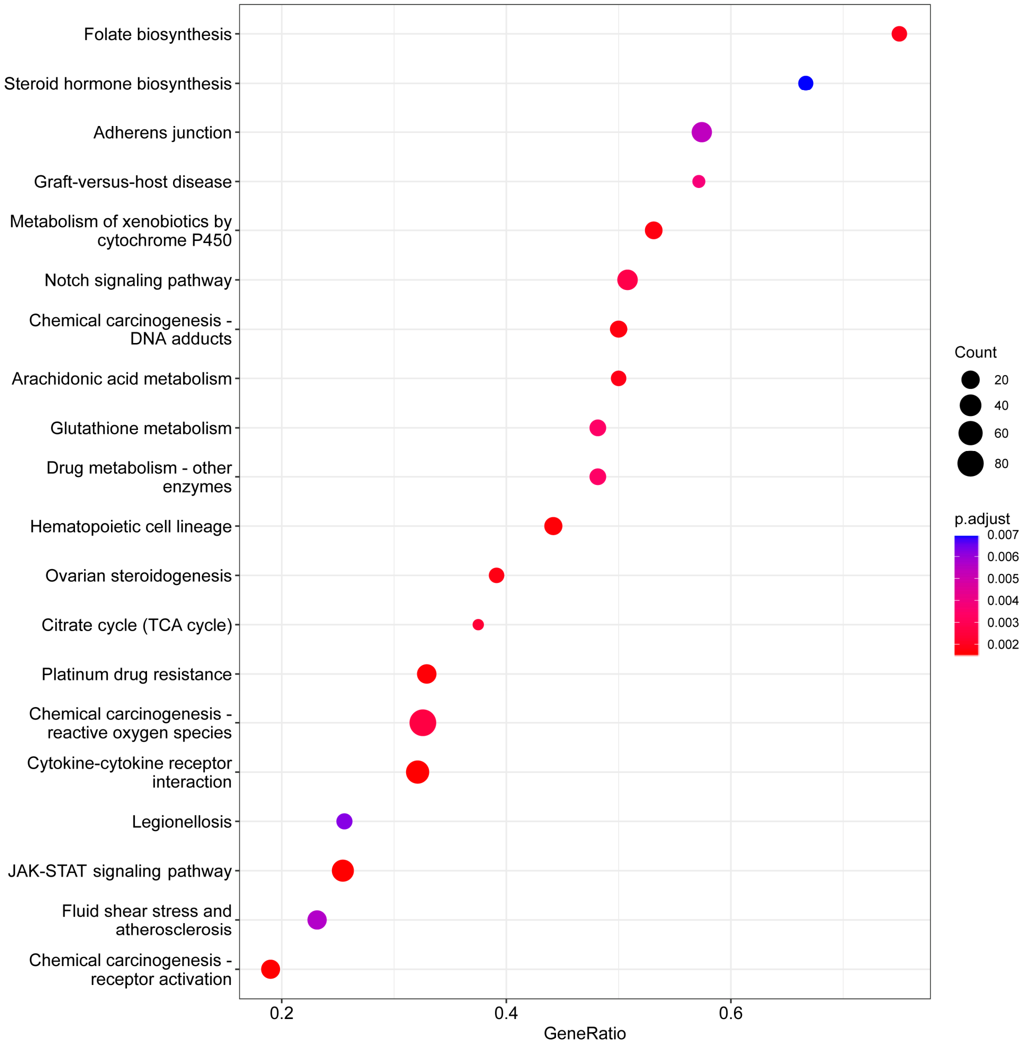

Figure S2. Dotplot of KEGG enrichment analysis of intestinal transcriptome in heat-stressed Hu sheep fed diets supplemented with rumen-protected taurine (RPT).

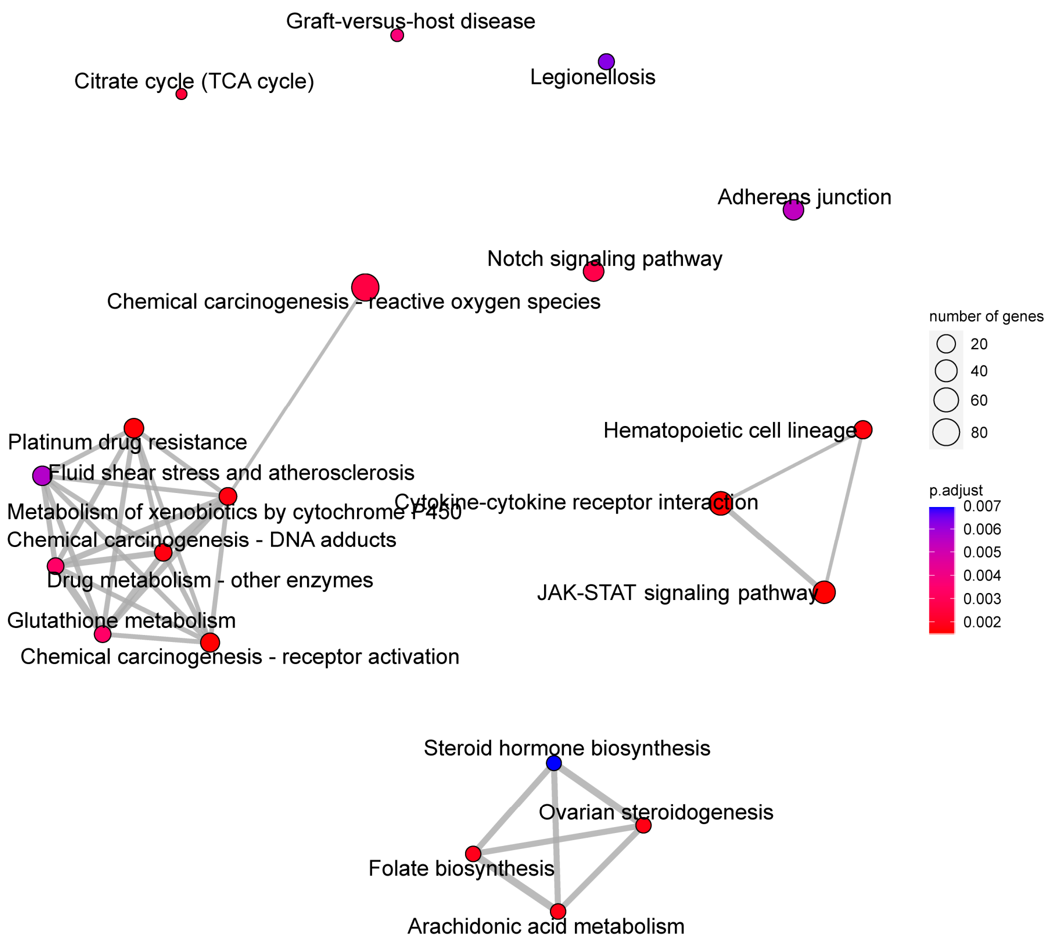

Figure S3. Kegg pathway network diagram focusing on inflammation-related pathways of intestinal transcriptome in heat-stressed Hu sheep fed diets supplemented with rumen-protected taurine (RPT).

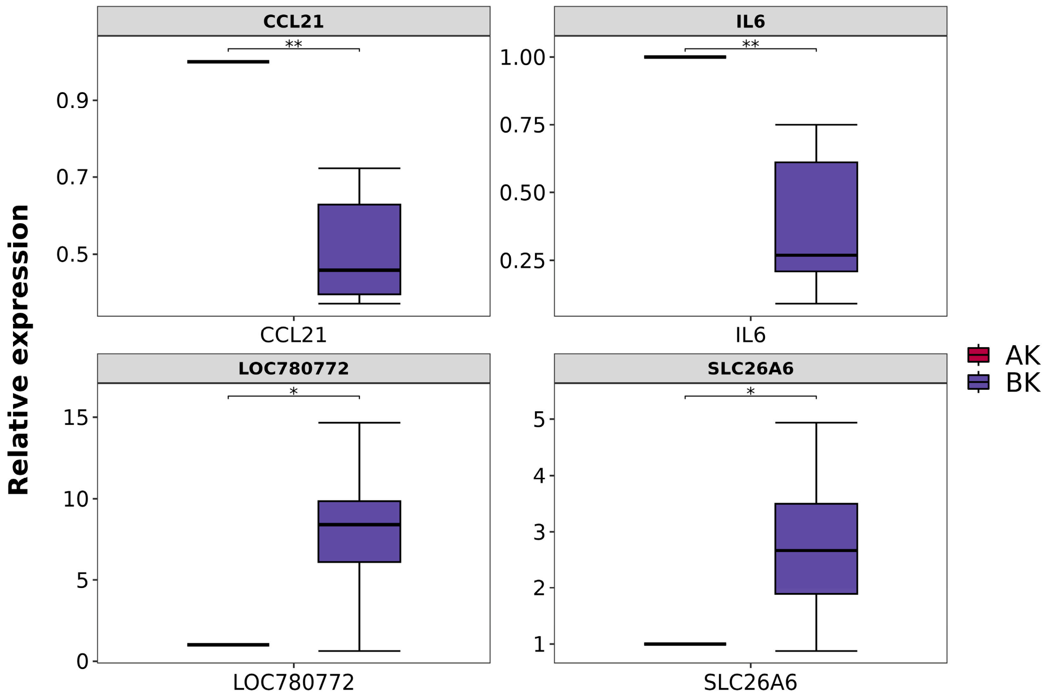

Figure S4. Boxplot of relative expression levels validating two upregulated and downregulated differentially expressed genes from the transcriptome using real-time quantitative PCR, AK represents the control group, BK represents the taurine-supplemented group (moderate level rumen-protected taurine, MT group), relative expression levels in the control group are all 1.

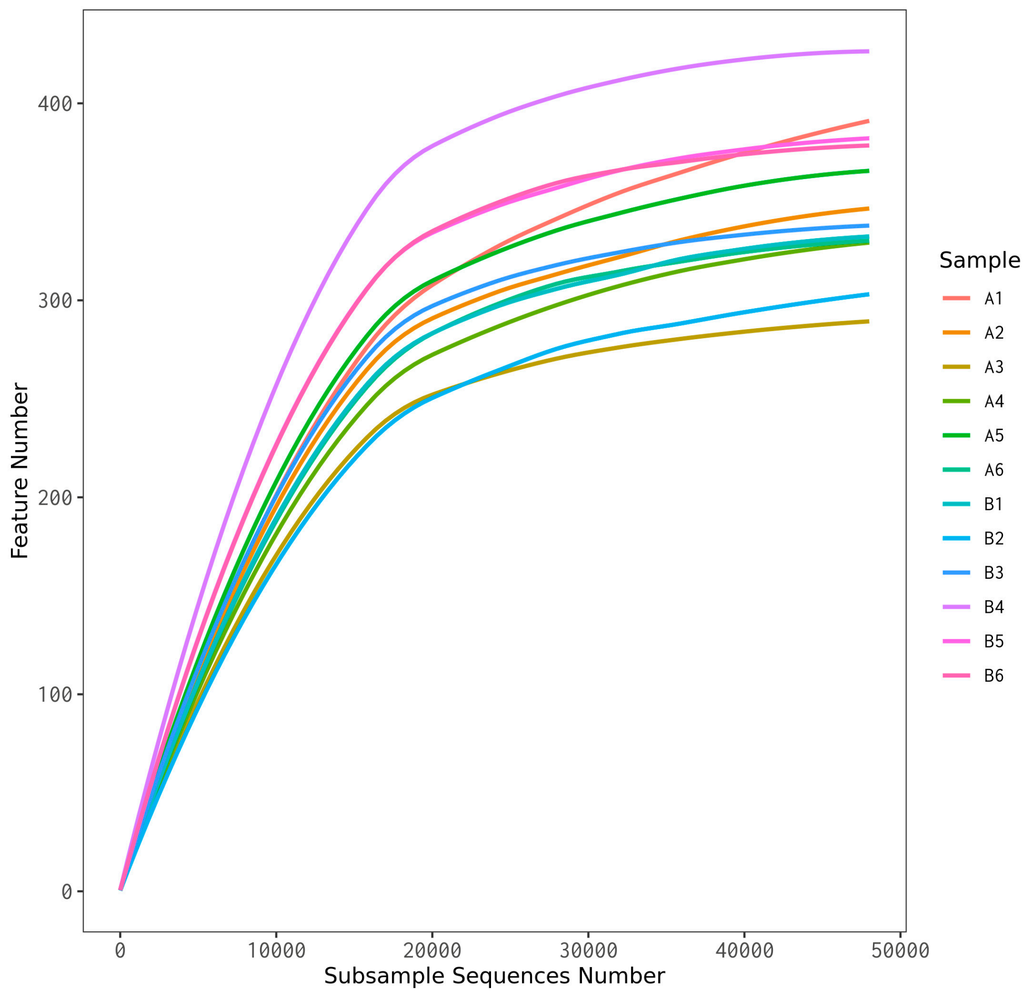

Figure S5. The rarefaction curve plot of microbial community in 12 samples of jejunal contents of Hu sheep. A1–A6 represent the six samples from the Control group, while B1–B6 represent the six samples from the Treatment (MT) group.

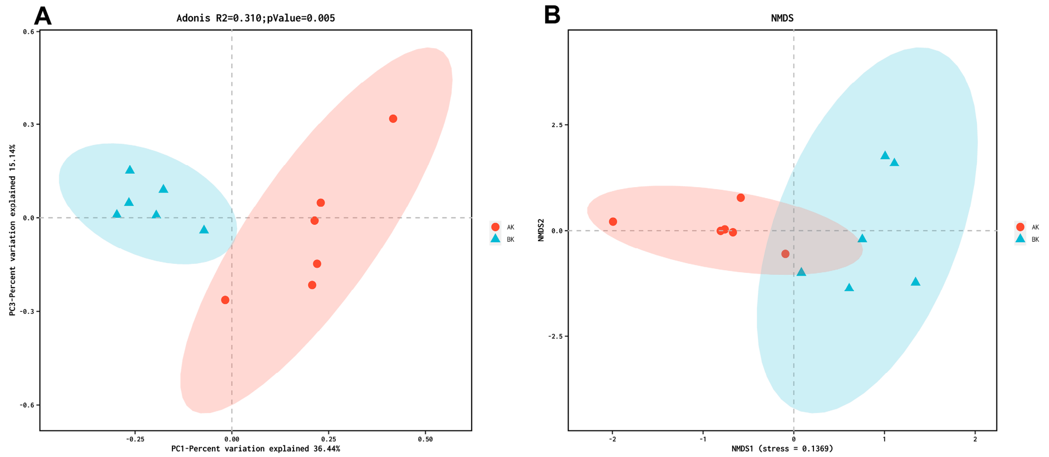

Figure S6. Profile of gut microbial β-diversity in heat-stressed Hu sheep fed diets supplemented with RPT. A, Principal coordinates analysis (PCoA) and B, Non-metric multidimensional scaling (NMDS) based on gut microbial β-diversity between the CON group (AK) and the MT group (BK).

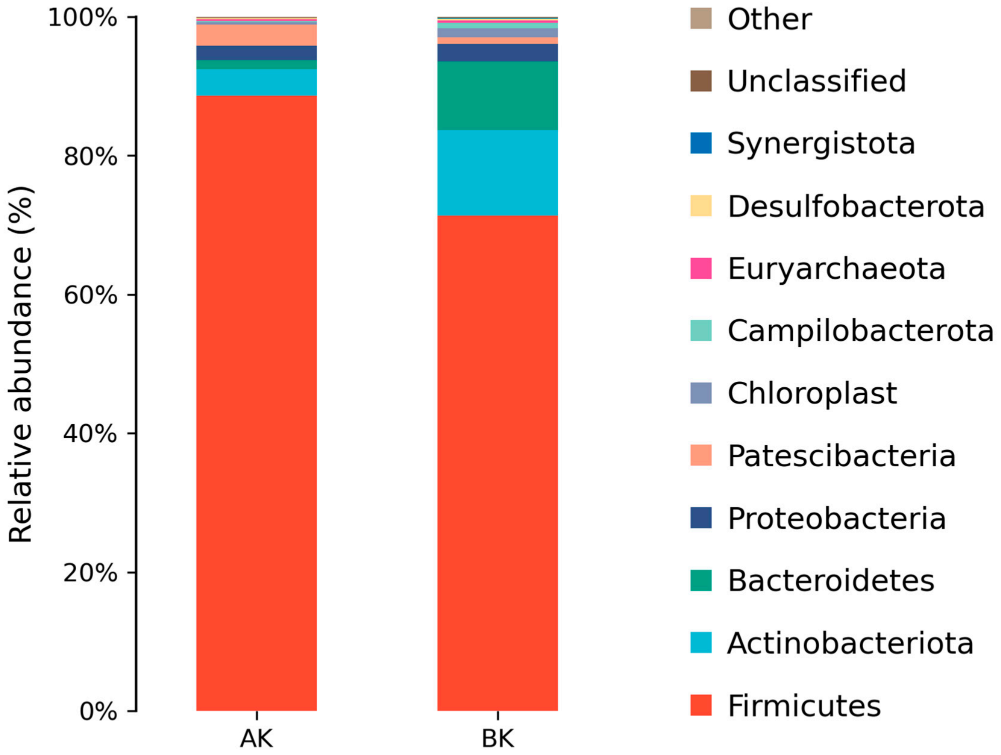

Figure S7. Comparison of intestinal microbiota at phylum level in heat-stressed Hu sheep, AK represents the control group, BK represents the taurine-supplemented group (moderate level rumen-protected taurine, MT group).

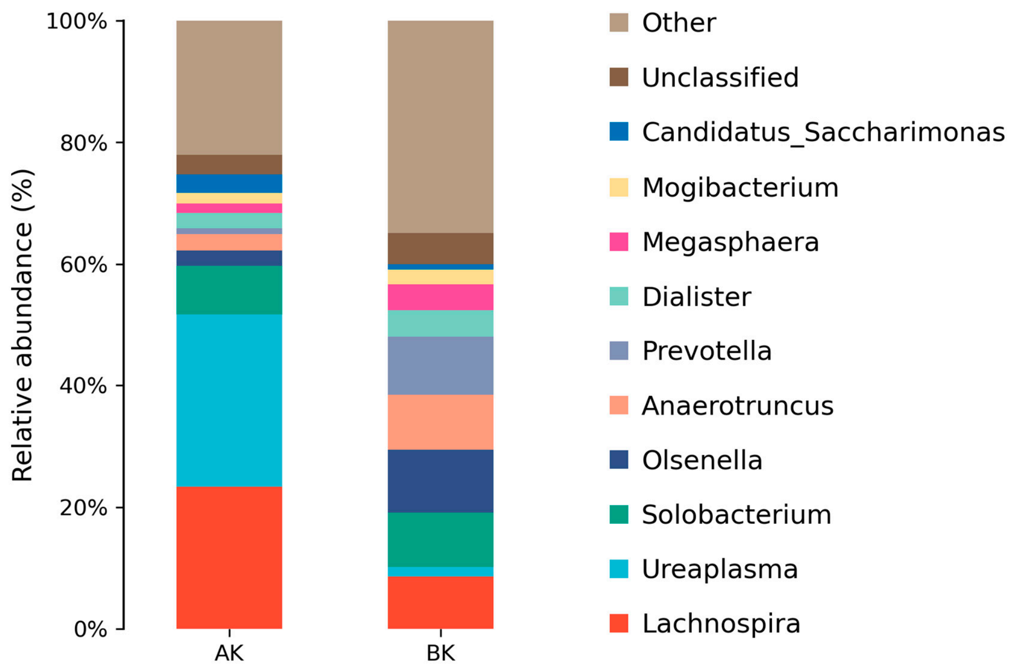

Figure S8. Comparison of intestinal microbiota at genus level in heat-stressed Hu sheep, AK represents the control group, BK represents the taurine-supplemented group (moderate level rumen-protected taurine, MT group).

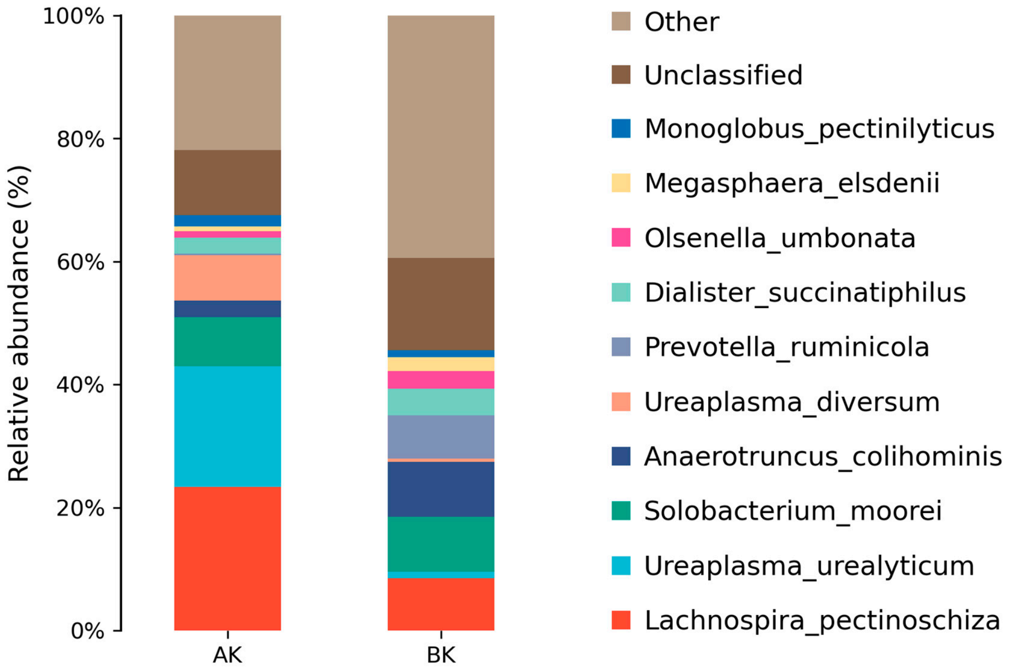

Figure S9. Comparison of intestinal microbiota at species level in heat-stressed Hu sheep, AK represents the control group, BK represents the taurine-supplemented group (moderate level rumen-protected taurine, MT group).

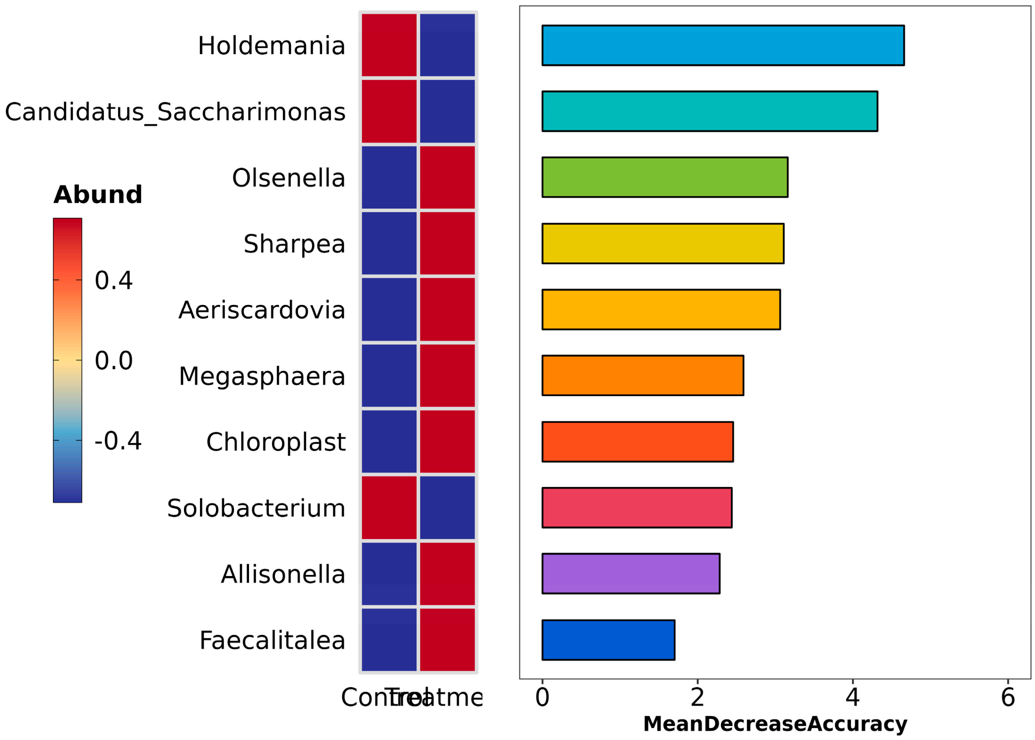

Figure S10. Random forest analysis of intestinal microbiota at genus level in heat-stressed Hu sheep.

Table S1. Primers for quantitative real-time PCR (*Ovis aries*).

Gene Name	GenBank accession	Primer sequences (5'-3')
CCL21	XM_004004119.5	F: GGACTGTTGCCTCACGTACA
		R: TCCGAGGCGAGAACAGGATA
IL6	NM_001009392.1	F: AATCTGGGTTCAATCAGGCGA
		R: TGCTCTGCAACTCCATGACA
LOC780772	NM_001093781.1	F: AGGATCGTGTGGAACACAGC
		R: AACCCTCTCACCAGTCCAGT
SLC26A6	XM_042236263.2	F: CACATCTCTGTGGGCACCTT
		R: TGACTGTGGAGTTCAAGCCC

Table S2. Clean read counts and reference genome alignment rates in the liver transcriptomes of 12 Hu sheep individuals

Samples ID [1]	Clean reads	Mapped rate
AK1	42,974,524	98.14
AK2	42,991,562	98.25
AK3	43,057,078	97.7
AK4	43,028,720	98.18
AK5	43,089,756	98.71
AK6	43,056,854	97.52
BK1	43,026,294	97.24
BK2	43,024,028	98.39
BK3	43,095,572	98.02
BK4	43,174,226	98.4
BK5	43,003,374	97.99
BK6	43,085,156	98.4

[1] AK1–AK6 represent the six samples from the control group with 0% RPT supplementation, while BK1–BK6 represent the six samples from the group supplemented with 0.4% RPT.

Table S3. Microbial sequences and α-diversity indices in jejunal contents of Hu sheep.

Samples [1]	Clean reads	Filtered reads	Mean length (bp)	Chao1	ACE	Shannon	Simpson
A1	145675	139618	1437	460.754	467.572	3.333	0.733
A2	72746	71127	1450	365.375	372.895	4.497	0.834
A3	63635	62148	1454	291.347	295.171	4.205	0.852
A4	73881	68355	1444	338.750	346.956	4.606	0.907
A5	71924	65829	1446	377.267	384.416	5.154	0.929
A6	65510	60223	1444	331.932	334.942	4.681	0.913
B1	65275	63739	1447	340.357	346.324	4.338	0.854
B2	72453	71403	1448	312.750	321.908	4.669	0.893
B3	56899	56008	1448	339.703	343.010	5.370	0.948
B4	53569	51744	1446	427.000	427.000	6.052	0.968
B5	64975	63606	1453	387.276	392.165	5.345	0.937
B6	54666	52588	1452	379.015	379.621	5.342	0.929

[1] A1–A6 represent the six samples from the control group with 0% RPT supplementation, while B1–B6 represent the six samples from the group supplemented with 0.4% RPT.
